# Behavior Change Techniques in Popular Alcohol Reduction Apps: Content Analysis

**DOI:** 10.2196/jmir.4060

**Published:** 2015-05-14

**Authors:** David Crane, Claire Garnett, James Brown, Robert West, Susan Michie

**Affiliations:** ^1^Department of Clinical, Educational and Health PsychologyUniversity College LondonLondonUnited Kingdom; ^2^Cancer Research UK Health Behaviour Research CentreUniversity College LondonLondonUnited Kingdom

**Keywords:** alcohol, behaviour change, mHealth, smartphone, iPhone, android, apps, digital, intervention

## Abstract

**Background:**

Mobile phone apps have the potential to reduce excessive alcohol consumption cost-effectively. Although hundreds of alcohol-related apps are available, there is little information about the behavior change techniques (BCTs) they contain, or the extent to which they are based on evidence or theory and how this relates to their popularity and user ratings.

**Objective:**

Our aim was to assess the proportion of popular alcohol-related apps available in the United Kingdom that focus on alcohol reduction, identify the BCTs they contain, and explore whether BCTs or the mention of theory or evidence is associated with app popularity and user ratings.

**Methods:**

We searched the iTunes and Google Play stores with the terms “alcohol” and “drink”, and the first 800 results were classified into alcohol reduction, entertainment, or blood alcohol content measurement. Of those classified as alcohol reduction, all free apps and the top 10 paid apps were coded for BCTs and for reference to evidence or theory. Measures of popularity and user ratings were extracted.

**Results:**

Of the 800 apps identified, 662 were unique. Of these, 13.7% (91/662) were classified as alcohol reduction (95% CI 11.3-16.6), 53.9% (357/662) entertainment (95% CI 50.1-57.7), 18.9% (125/662) blood alcohol content measurement (95% CI 16.1-22.0) and 13.4% (89/662) other (95% CI 11.1-16.3). The 51 free alcohol reduction apps and the top 10 paid apps contained a mean of 3.6 BCTs (SD 3.4), with approximately 12% (7/61) not including any BCTs. The BCTs used most often were “facilitate self-recording” (54%, 33/61), “provide information on consequences of excessive alcohol use and drinking cessation” (43%, 26/61), “provide feedback on performance” (41%, 25/61), “give options for additional and later support” (25%, 15/61) and “offer/direct towards appropriate written materials” (23%, 14/61). These apps also rarely included any of the 22 BCTs frequently used in other health behavior change interventions (mean 2.46, SD 2.06). Evidence was mentioned by 16.4% of apps, and theory was not mentioned by any app. Multivariable regression showed that apps including advice on environmental restructuring were associated with lower user ratings (Β=-46.61, *P*=.04, 95% CI -91.77 to -1.45) and that both the techniques of “advise on/facilitate the use of social support” (Β=2549.21, *P*=.04, 95% CI 96.75-5001.67) and the mention of evidence (Β=1376.74, *P*=.02, 95%, CI 208.62-2544.86) were associated with the popularity of the app.

**Conclusions:**

Only a minority of alcohol-related apps promoted health while the majority implicitly or explicitly promoted the use of alcohol. Alcohol-related apps that promoted health contained few BCTs and none referred to theory. The mention of evidence was associated with more popular apps, but popularity and user ratings were only weakly associated with the BCT content.

## Introduction

Excessive alcohol use causes approximately 3.3 million deaths each year, and more than 5% of the global burden of disease is attributable to its consumption [1]. Brief interventions delivered by health care workers to hazardous drinkers are effective [2]. However, there is little understanding of their mechanisms of action [3], and barriers to their delivery such as lack of time, training, and financial resources mean they have limited reach [4,5].

Mobile phones offer the potential to reduce these barriers as they are relatively cheap, accessible to users, and deliver support when and where needed. Mobile phone apps for mobile health (mHealth) enable disease monitoring, management, and education; improve health behavior assessment; and facilitate more frequent user-to-user or practitioner-to-user communication [6-8]. Thousands of mHealth apps are available; as of June 2013, there were over 40,000 mHealth apps in the US English-language iTunes Store alone [9]. Approximately 20% of smartphone users have downloaded an mHealth app [10]; this figure is expected to rise as both smartphone ownership and the number of apps increase. According to industry estimates, 1.7 billion smartphone users worldwide will have downloaded an mHealth app by 2018 [11].

Smartphone use is increasing rapidly among young people, but older people are also using apps in increasing numbers [12]. App-delivered interventions to reduce excessive alcohol consumption could potentially be targeted at a range of age groups, as younger people tend to drink more heavily but older people tend to drink more regularly [1].

Despite the proliferation of mHealth apps, there has been little research investigating their mechanisms of action or efficacy and they are often developed without reference to evidence base or theory [13]. Reviews of apps for smoking cessation [14], weight loss [15-17], diabetes management [18], healthy eating and physical activity [19,20], breast disease management [21], and melanoma detection [22] have found the majority fail to conform to guidelines, lack evidence-based content, and/or provide inaccurate information. Concern about the content of mHealth apps has led to calls for regulation to improve patient safety [23,24].

Moreover, the most popular apps—as defined by the approximate number of installations on the Google Play store or by their position in the search results in the iTunes Store—have been found to contain fewer evidence-based techniques [15], lower levels of adherence to guidelines [14], or information that may encourage risky behavior [25] than less popular apps. User ratings are a different measure of popularity and reflect a user’s judgment about the quality of the app (eg, an app may be highly rated but used by only a small number of people). User ratings have been found to be associated with high levels of adherence to guidelines in smoking cessation apps [14], although not in weight loss [17] or physical activity apps [20].

A review of 767 alcohol apps available in the US version of the iTunes Store found that 71% facilitated the use of alcohol and 29% aimed to reduce its consumption [26], though many of the alcohol reduction apps simply attempted to measure a user’s blood alcohol content (BAC). A review of 384 BAC apps available in the Australian iTunes and Google Play stores found that most were inaccurate, with some purporting to measure BAC by asking users to blow into the microphone, and only 11% of all the apps examined had an alcohol reduction focus [25].

Of the US and Australian alcohol-reduction apps identified in previous studies [25,26], little is known about their potential active ingredients and mechanisms of action. A useful method for describing the potentially active ingredients of apps is to assess the behavior change techniques (BCTs) they contain [27-29]. A BCT is “an observable, replicable, and irreducible component of an intervention designed to alter or redirect causal processes that regulate behavior; that is, a technique is proposed to be an ‘active ingredient’ (eg, feedback, self-monitoring, and reinforcement)” (p. 82, [27]).

A taxonomy of 42 BCTs to reduce excessive alcohol consumption has been developed from guidance documents and treatment manuals identified by expert consultation [30]. The taxonomy has been reliably applied to identifying BCTs in brief alcohol interventions, and meta-regression revealed that those that included self-monitoring were associated with larger effect sizes [30]. Similar taxonomies have been used to reliably identify BCTs contained in physical activity and dietary apps [31,32].

An additional aim of this study was to identify whether there were BCTs widely used in domains other than alcohol consumption that could be considered for alcohol apps. Analysis of the BCTs used in 40 published descriptions of behavior change interventions to prevent illness, improve illness management, or improve the behaviors of health care professionals found that 22 of a possible 93 BCTs were frequently used. A comparison with those used in alcohol apps would indicate potentially useful BCTs not frequently used in alcohol apps.

The current study should yield benefits for research and practice. Coding alcohol apps for BCTs allows (1) researchers to identify BCTs and establish which ones are based on theory and/or evidence and to conduct evaluations in terms of BCTs, (2) users to be better informed about which BCTs are present and enable them to choose ones suited to their needs, (3) health care practitioners to make more informed recommendations to patients [33], and (4) app developers to make decisions about which BCTs to include.

This study builds on previous work [25,26] by providing an up-to-date estimate of the relative prevalence of alcohol-related apps available in the United Kingdom that focus on reducing excessive alcohol consumption and by coding those apps for their component BCTs [30]. We also explored associations between the presence of BCTs, the mention of theory or evidence, and the popularity and user ratings of the app.

The research questions addressed by this study are (1) What proportion of alcohol-related apps available in the UK version of the iTunes and Google Play stores focus on alcohol reduction?, (2) Which BCTs are contained within alcohol-related apps focusing on alcohol reduction?, (3) To what extent do alcohol-related apps focusing on alcohol reduction use BCTs commonly found in other types of behavior change intervention?, and (4) What are the associations between the presence of BCTs, the mention of theory or evidence, and the popularity and user ratings of the apps?

## Methods

### Search Strategy and Data Extraction

Alcohol-related apps were identified by searching the UK versions of the iTunes and Google Play stores in April and May 2014 for the terms “alcohol” and “drink”. The following data were extracted from the first 200 results found for each term in each app store (4 x 200): time and location of search, app name, developer name, ranking in the search results, cost, and classification. We considered 200 search results for each search term comprehensive as users rarely examine search results thoroughly [34].

Duplicate apps were removed from the 800 search results and the unique apps were classified as either alcohol reduction (apps that aim to reduce drinking-related behavior and those that track consumption), entertainment (drinking games, cocktail recipes, bar finders); BAC measurement; or other (apps not about alcohol, apps not in English, information for employers, etc).

**Figure 1:**
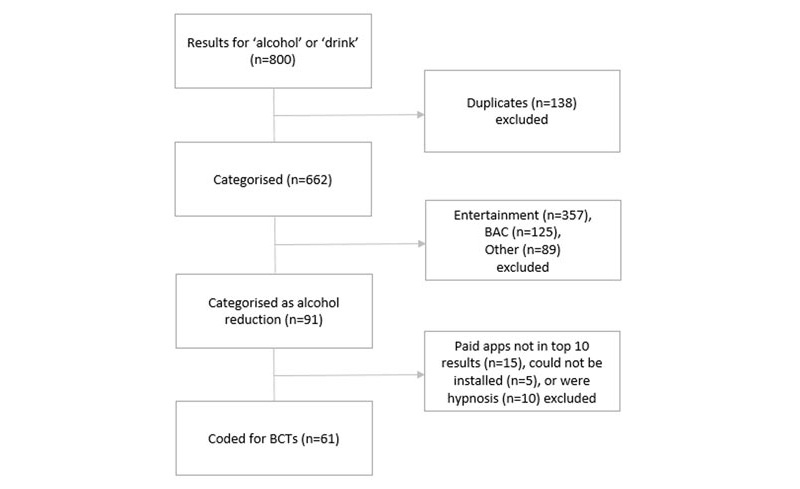
Flow diagram of apps selected for coding.

Of the 91 alcohol reduction apps, we installed, examined, and coded all 51 free apps as users prefer apps that are free to download [35]. However, 10 paid apps were installed, examined, and coded as a sensitivity check of the BCTs included. The remaining paid apps (n=15), apps that could not be installed (n=5), or those that focused on hypnosis (n=10) were excluded (see Figure 1). Included apps were coded for the presence of BCTs [30], mention of theory, mention of evidence, number of installations, and user ratings. Ratings were taken from all versions of the app in the iTunes store (rather than the current version). We did not base our coding on any other information (such as descriptions in the app stores or on Web pages, or within developers’ protocols or published papers).

### Behavior Change Technique Coding

The taxonomy of BCTs to reduce excessive alcohol consumption and the associated coding manual were used for our evaluation [30]. The coding manual includes definitions and examples of BCTs and guidelines for identifying them in intervention descriptions. For each app, BCTs were coded as 0: no evidence of BCT, 1: BCT present in all probability but evidence unclear; and 2: BCT present beyond all reasonable doubt and clear evidence. For all analyses, the presence of a BCT was dichotomized, with only those receiving a “2” being classified as containing the BCT. The BCT “build general rapport” was excluded from coding because it could not be appropriately coded for a digital intervention.

The coding manual was used independently by 2 trained coders (DC and CG) to code 11 of the included apps. There was “outstanding” agreement: prevalance and bias adjusted kappa (PABAK)=.89, kappa=.65 [36] for this first round of coding. Discrepancies were discussed and the coding guidelines were refined. After the coding manual was updated, the remaining apps were coded by 1 coder (DC) with 15% also coded by the second coder (CG) to assess rater drift. There was “outstanding” agreement for the subsequent ratings of the eight apps coded by both raters (PABAK=.89, kappa=.81 [36]).

Of the 93 BCTs described by a general taxonomy of behavior change (BCTTv1) [27], 22 have been found to be frequently used in a variety of health behavior change interventions [37]. In order to establish the extent to which the included apps contained these BCTs, 1 coder (DC) and an independent behavior change expert mapped the 22 frequently used BCTs to the alcohol taxonomy [30]. This allowed us to determine the prevalence of those BCTs in these apps.

The full content of the apps was coded. Alcohol consumption spanning numerous days was entered in order to determine if the app included graphs that displayed progress over time. If the app was tailored on the basis of personal details, the characteristics of a female alcohol consumer in the United Kingdom drinking moderately above guidelines were used (30 years, consumed 16 units of alcohol over 3 days in the previous week). Theory was coded if the app made reference to theory as a factor informing its development. Evidence was coded if the app made reference to empirical evidence relating to behavior change. Apps were coded on an iPhone running iOS7 and a Samsung Galaxy S3 running Android 4.3.

### Popularity and User Ratings

The popularity of apps was operationalized as the overall number of ratings received. User ratings of the apps were operationalized by assessing the proportion of ratings that were four or five star and calculating the associated lower 95% confidence interval (CI). Mean ratings were not used because they do not reflect the uncertainty associated with a very small number of ratings [38]. For example, an app with 2 five-star reviews and no other reviews would receive a mean rating of 5 and an app with 900 five-star reviews and 100 one-star reviews would receive a mean rating of 4.6. Whereas, if using a lower bound CI, an app with 2 five-star reviews would have a lower bound CI of .34, and an app with 900 five-star reviews and 100 one-star reviews would have a lower bound CI of .88. This approach has been adopted by websites such as reddit and Yelp, which depend on accurately ranking user ratings [39,40].

### Analyses

All statistical analyses were conducted using SPSS version 20.0. Frequencies, percentages, and associated 95% CIs were calculated for the categories of alcohol-related apps (alcohol reduction, entertainment, blood alcohol content, other), for each of the 41 BCTs, and for the mention of theory or the mention of evidence contained within the alcohol reduction apps. To assess interrater reliability, kappa and PABAK were calculated. PABAK is an adjusted kappa statistic that accounts for coders agreeing on the presence or the absence of codes [41]. PABAK was used in addition to Cohen’s kappa, which only accounts for coders agreeing on the presence of codes*.*

We examined (1) all BCTs in Table 1 for their frequency in alcohol reduction apps, (2) associations between the presence of BCTs and the mention of theory or evidence listed in Table 2 with the popularity of the app in a series of univariable logistic regressions, and (3) the independent association after mutual adjustment for all variables listed in Table 2 in a multivariable logistic regression. BCTs that were present in two or fewer apps were excluded. We repeated similar analyses to examine the univariable and multivariable associations between the presence of BCTs and the mention of theory or evidence listed in Table 3 with the user ratings.

## Results

### Overview

Of the 800 apps returned from the searches, 662 unique apps were identified (see Figure 1). Of these, 13.7% were classified as alcohol reduction (91/662, 95% CI 11.3-16.6), 53.9% entertainment (357/662, 95% CI 50.1-57.7), 18.9% blood alcohol content measurement (125/662, 95% CI 16.1-22.0), and 13.4% other (89/662, 95% CI 11.1-16.3). A total of 61 apps were coded: all 51 free apps and the first 10 paid apps found in the search results. The remaining paid apps (n=15), apps that could not be installed (n=5), and those that focused on hypnosis (n=10) were excluded.

### Behavior Change Techniques in Alcohol Reduction Apps

A sensitivity check indicated that the number and type of BCTs in free and paid apps was broadly similar, so they were treated as a single group (data not shown). The mean number of BCTs from the alcohol taxonomy [30] used in apps was 3.56 (SD 3.39, median 2). Seven apps did not include any BCTs and 30 apps (49.2%) included only one, two, or three BCTs. Five apps included 10 or more BCTs. The maximum number of BCTs included was 13 (n=3), and 26 BCTs were used in at least one app.

The frequency with which BCTs were included by different apps is shown in Table 1. The most frequent BCTs were “facilitate self-recording” (54.1%, 33/61), “provide information on consequences of excessive alcohol use and drinking cessation” (42.6%, 26/61), “provide feedback on performance” (41.0%, 25/61), “give options for additional and later support” (24.6%, 15/61), and “offer/direct towards appropriate written materials” (23.0%, 14/61).

**Table 1:** BCTs included in alcohol reduction apps (N=61 apps).

BCT		n (%)
15	Facilitate self-recording	33 (54.1)
1	Provide information on consequences of excessive alcohol use and drinking cessation	26 (42.6)
3	Provide feedback on performance	25 (41.0)
22	Give options for additional and later support	15 (24.6)
32	Offer/direct towards appropriate written materials	14 (23.0)
23	Tailor interactions appropriately	13 (21.3)
2	Boost motivation and self‐efficacy	9 (14.8)
14	Prompt review of goals	8 (13.1)
4	Provide rewards contingent on successfully reducing excessive alcohol use/abstaining	8 (13.1)
13	Facilitate goal setting	7 (11.5)
33	Provide information on withdrawal symptoms	6 (9.8)
17	Advise on environmental restructuring	5 (8.2)
42	Behavior substitution	5 (8.2)
10	Facilitate barrier identification and problem solving	4 (6.6)
11	Facilitate relapse prevention and coping	4 (6.6)
20	Advise on avoidance of social cues for drinking	4 (6.6)
21	Advise on/facilitate use of social support	4 (6.6)
6	Prompt commitment from the user there and then	4 (6.6)
12	Facilitate action planning/know how to help identify relapse triggers	3 (4.9)
25	Assess current and past drinking behavior	3 (4.9)
5	Provide normative information about others’ behavior and experiences	3 (4.9)
16	Change routine	2 (3.3)
24	Emphasize choice	2 (3.3)
37	Provide reassurance	2 (3.3)
7	Provide rewards contingent on effort or progress	2 (3.3)
8	Identify reasons for wanting and not wanting to reduce excessive alcohol use	2 (3.3)
18	Set graded tasks	1 (1.6)
26	Assess current readiness and ability to reduce excessive alcohol use	1 (1.6)
31	Explain expectations regarding treatment programme	1 (1.6)
9	Explain the importance of abrupt cessation	1 (1.6)
19	Advise on conserving mental resources	0 (0)
27	Assess past history of attempts to reduce excessive alcohol use	0 (0)
28	Assess withdrawal symptoms	0 (0)
30	Elicit and answer questions	0 (0)
34	Use reflective listening	0 (0)
35	Elicit user views	0 (0)
36	Summarize information/confirm user decisions	0 (0)
38	Model/demonstrate the behavior	0 (0)
39	Prompt use of imagery	0 (0)
40	Motivational interviewing	0 (0)
41	General communication skills training	0 (0)

Eleven BCTs were not used in any app: “advise on conserving mental resources”, “assess past history of attempts to reduce excessive alcohol use”, “assess withdrawal symptoms”, “elicit and answer questions”, “use reflective listening”, “elicit user views”, “summarize information/confirm user decisions”, “model/demonstrate the behavior”, “prompt use of imagery”, “motivational interviewing”, and “general communication skills training”.

### Behavior Change Techniques Frequently Found in Other Interventions and Used in Alcohol Apps

Of the 22 BCTs frequently found in other health behavior change interventions, the mean number included in alcohol-reduction apps was 2.46 (SD 2.06, median 2). Of these 22, the five most often included in alcohol apps were “facilitate self-recording” (54.1%, 33/61), “provide information on consequences of excessive alcohol use and drinking cessation” (42.6%, 26/61), “provide feedback on performance” (41.0%, 25/61), “give options for additional and later support” (24.6%, 15/61), and “offer/direct towards appropriate written materials” (23.0%, 14/61). Three of the BCTs frequently found in other health behavior change interventions were not used in any app “motivational interviewing”, “use reflective listening”, and “model/demonstrate the behavior”.

### Associations Between Behavior Change Techniques, Theory, and Evidence With Popularity and User Ratings

The mean user rating for apps was 2.64 (SD 1.71), and the mean number of ratings was 234.46 (SD 1272.08). Evidence was mentioned in 16.4% of apps (n=10), most usually evidence about the recommended guidelines for the consumption of alcohol. Theory was not mentioned by any app.

The BCT “prompt review of goals” (B=0.41, *P*=.001, 95% CI 11.88-44.79) was positively associated with user ratings in univariable regression models (Table 2); no other significant associations between BCTs and user ratings were found. In multivariable linear regression models, the only significant association was a negative one: apps that advised on environmental restructuring had marginally lower user ratings (Β=-46.61, *P*=.04, 95% CI -91.77 to -1.45).

**Table 2:** The association between BCTs, theory/evidence with ratings (lower 95% CI of the proportion of ratings >3/5).^a^

BCT		Unadjusted Β (CI)	Adjusted B (CI)
1	Provide information on consequences of excessive alcohol use and drinking cessation	0.08 (-8.59 to 15.96)	-6.54 (-32.64 to 19.56)
2	Boost motivation and self‐efficacy	0.13 (-8.78 to 25.29)	17.88 (-9.77 to 45.53)
3	Provide feedback on performance	0.23 (-1.25 to 22.86)	-14.28 (-43.21 to 14.65)
4	Provide rewards contingent on successfully reducing excessive alcohol use/abstaining	0.18 (-5.25 to 30.23)	4.73 (-25.16 to 34.62)
6	Prompt commitment from the user there and then	0.18 (-7.21 to 41.18)	-31.96 (-83.87 to 19.94)
10	Facilitate barrier identification and problem solving	-0.03 (-27.15 to 22.03)	-62.14 (-139.39 to 15.12)
11	Facilitate relapse prevention and coping	-0.17 (-39.96 to 8.55)	-17.62 (-94.96 to 59.71)
13	Facilitate goal setting	0.19 (-4.96 to 32.57)	15.19 (-16.26 to 46.64)
14	Prompt review of goals	0.41 (11.88 to 44.79)^b^	24.34 (-3.67 to 52.34)
15	Facilitate self-recording	0.17 (-4.01 to 20.07)	-0.92 (-27.75 to 25.91)
17	Advise on environmental restructuring	-0.1 (-30.69 to 13.48)	-46.61 (-91.77 to -1.45)^b^
20	Advise on avoidance of social cues for drinking	0.06 (-18.82 to 30.28)	18.98 (-38.64 to 76.61)
21	Advise on/facilitate use of social support	0.05 (-19.66 to 29.46)	2.39 (-42.95 to 47.73)
22	Give options for additional and later support	0.05 (-11.55 to 16.7)	-2.04 (-44.97 to 40.89)
23	Tailor interactions appropriately	0.23 (-1.16 to 27.76)	-0.89 (-26.32 to 24.54)
32	Offer/direct towards appropriate written materials	0.02 (-13.51 to 15.44)	-16.25 (-50.57 to 18.07)
33	Provide information on withdrawal symptoms	-0.06 (-25.39 to 15.42)	-6.91 (-54.04 to 40.22)
42	Behavior substitution	-0.07 (-27.65 to 16.65)	-5.25 (-64.82 to 54.32)
	Total BCTs	0.16 (-0.66 to 2.91)	6.29 (-13.28 to 25.87)
	Mention of evidence	0.22 (-2.25 to 29.85)	18.15 (-3.45 to 39.74)

^a^BCTs only included for analysis if present in more than two apps. The adjusted models included all variables listed in this table.

^b^Indicates *P*<.05.

The mention of evidence (B=0.26, *P*=.04, 95% CI 24.28-1739.31) was positively associated with the popularity of the apps in univariable regression models (Table 3). In a multivariable linear regression models, both “advise on/facilitate the use of social support” (Β=2549.21, *P*=.04, 95% CI 96.75-5001.67) and the mention of evidence (Β=1376.74, *P*=.02, 95% CI 208.62-2544.86) were positively associated with popularity.

**Table 3:** The association between BCTs, theory/evidence with popularity (number of ratings).

BCT		Unadjusted B (CI)	Adjusted B (CI)
1	Provide information on consequences of excessive alcohol use and drinking cessation	0.19 (-155.79 to 1148.02)	906.92 (-504.77 to 2318.61)
2	Boost motivation and self-efficacy	-0.06 (-1143.07 to 706.91)	-228.05 (-1723.82 to 1267.72)
3	Provide feedback on performance	0.2 (-138.23 to 1170.96)	410.01 (-1154.91 to 1974.93)
4	Provide rewards contingent on successfully reducing excessive alcohol use/abstaining	-0.03 (-1101.77 to 844.34)	-1362.93 (-2979.54 to 253.69)
6	Prompt commitment from the user there and then	-0.05 (-1563.56 to 1089.02)	-644.43 (-3452.1 to 2163.25)
10	Facilitate barrier identification and problem solving	-0.05 (-1570.16 to 1082.25)	-2150.59 (-6329.64 to 2028.46)
11	Facilitate relapse prevention and coping	-0.05 (-1574.65 to 1077.64)	2175.79 (-2007.23 to 6358.81)
13	Facilitate goal setting	0.05 (-849.23 to 1210.96)	828.87 (-872.37 to 2530.11)
14	Prompt review of goals	0.04 (-838.87 to 1107.13)	-751.26 (-2266.15 to 763.63)
15	Facilitate self-recording	0.15 (-264.74 to 1038.84)	547.11 (-904.17 to 1998.39)
17	Advise on environmental restructuring	-0.05 (-1441.99 to 950.81)	-1189.63 (-3632.18 to 1252.92)
20	Advise on avoidance of social cues for drinking	-0.05 (-1564.35 to 1088.21)	-2799.6 (-5916.7 to 317.49)
21	Advise on/facilitate use of social support	-0.05 (-1562.23 to 1090.37)	2549.21 (96.75 to 5001.67)^b^
22	Give options for additional and later support	0.2 (-149.92 to 1344.47)	-61.18 (-2383.46 to 2261.1)
23	Tailor interactions appropriately	-0.06 (-984.06 to 618.26)	-778.78 (-2154.21 to 596.65)
32	Offer/direct towards appropriate written materials	0.22 (-115.64 to 1410.65)	666.27 (-1190.03 to 2522.58)
33	Provide information on withdrawal symptoms	-0.06 (-1355.21 to 848.07)	-1868.23 (-4417.4 to 680.94)
42	Behavior substitution	-0.05 (-1440.49 to 952.36)	-1442.94 (-4665.2 to 1779.31)
	Total BCTs	0.07 (-70.01 to 124.48)	150.73 (-908.13 to 1209.58)
	Mention of evidence	0.26 (24.28 to 1739.31)^b^	1376.74 (208.62 to 2544.86)^b^

^a^BCTs only included for analysis if present in more than two apps. The adjusted models included all variables listed in this table.

^b^Indicates *P*<.05.

## Discussion

### Principal Findings

A review of 662 alcohol-related apps in the UK version of the iTunes and Google Play stores found that more than half were classified as entertainment apps that promoted drinking, 19% were BAC calculators, and 14% had an alcohol reduction focus. This is consistent with findings on alcohol-related apps available in the United States and Australian app stores [25,26] and indicates that potential app users who search for terms such as “alcohol” will be primarily exposed to apps encouraging increased alcohol consumption.

The BCTs most often used in alcohol reduction alcohol apps were (1) “facilitate self-recording” (included in 54% of apps), (2) “provide information on consequences of excessive alcohol use” (43%), (3) “provide feedback on performance” (41%), (4) “give options for additional and later support” (25%), and (5) “offer/direct towards appropriate written materials” (23%). The second, fourth, and fifth of these are information-based. This finding may indicate a missed opportunity for app developers, as interventions that require interaction from participants are associated with increased amounts of behavior change than interventions that passively present information [42].

Behavior change interventions are often complex and consist of a number of BCTs [43], which may interact additively or synergistically. For example, Control Theory [44] posits that goal-setting, feedback/self-monitoring, action planning, and goal review have synergistic effects. Interventions using a group of these techniques have been found to be more effective than interventions that used only one [45-47]. In alcohol reduction apps, “facilitate self-recording” and “provide feedback on performance” were found to be frequently used BCTs. However, other theory-linked BCTs were often not included: “prompt review of goals” was used in only 13% of apps, “facilitate goal setting” in 12%, and “facilitate action planning” in 5%.

The number of apps prompting the review of behavioral goals was greater than the number that facilitated goal setting, as in many cases apps assumed a user’s behavioral goal was to get their drinking below recommended daily or weekly guidelines and displayed a graph to indicate how current levels of drinking compared to guidelines. Apps that facilitated goal setting allowed users to set their own goals, for example, to have a set number of non-drinking days each week. People are motivated by different types of goals [48] and self-set goals tend to result in greater commitment to goal achievement than assigned goals [49]. Together these studies suggest that apps that allow users to set their own goals and review their performance against them would be more successful, but only three apps met this criteria.

The mean number of BCTs from the alcohol taxonomy [30] included in the reviewed apps was less than four. Five apps included more than 10 BCTs, three of which were book or pamphlet-type apps that passively provided information or advice. More BCTs does not necessarily equate to more effective interventions; interventions that targeted lower-income groups to reduce smoking or increase physical activity and/or healthy eating were found to be more effective when they contained fewer BCTs [50]. Other reviews have found a positive relationship between the number of BCTs and weight loss [45] and that health behavior change interventions that included more BCTs tended to have larger effect sizes [51].

The relatively low number of BCTs used in the majority of apps in this study suggests there is scope to investigate whether including more BCTs could increase effectiveness and additionally, whether BCTs found to be effective when delivered face-to-face could be effective when delivered digitally. For example, “provide normative information about others’ behavior and experiences” has been found effective in reducing alcohol consumption when delivered digitally [52,53] but was used in less than 5% of the apps reviewed in the current study. “Motivational interviewing” is another frequently used BCT and has been used in a Web-based intervention to reduce alcohol consumption [54] indicating the possibility for it to be delivered digitally, but no apps included this technique. It will be important to establish whether any BCTs found to be effective in other modes of delivery generalize to app-based interventions.

The 22 BCTs frequently found in other health behavior change interventions [37] were rarely used in alcohol apps (mean 2.5). Social support is the BCT most frequently found in other interventions but was only used in 7% of alcohol apps. “Facilitate action planning” is a frequently used BCT in other interventions but was included in less than 5% of apps. Action planning has been found effective when combined with feedback [46], but none of the apps in this study included both techniques. This finding suggests that developers of alcohol apps may benefit from looking across other domains. In doing so, it is useful to draw on theory to guide the selection of BCTs for any given intervention.

The BCT “prompt review of goals” was positively associated with user ratings in univariable models, and “advise on environmental restructuring” was negatively associated with user ratings in multivariable models. The mention of evidence was positively associated with the popularity of the app in univariable models, and both the mention of evidence and “advise on/facilitate the use of social support” were positively associated with popularity in multivariable models. Apps that mentioned evidence usually referred to evidence relating to the recommended guidelines for consumption rather than evidence about the approach to behavior change adopted by the app. No app mentioned theory.

The current study provided relatively little evidence of association between BCTs, mention of theory or evidence, and the popularity or user ratings of apps. However, the failure to identify evidence of associations should not be taken as evidence that there are not true associations. The relatively small number of alcohol reduction apps available for analysis meant the study was exploratory and had only limited power.

It may be that other BCTs are associated with user ratings and popularity, but the large variation in the design, complexity, and functionality of apps and the contexts in which use occurs may be masking such associations [55]. An app with a large number of BCTs could be poorly built and so result in a poor user experience, negative ratings, and few downloads, whereas an app with few BCTs could be well built and result in a good user experience, positive ratings, and increased downloads. Careful experimental work in factorial designs is required to isolate and test the impact of BCTs and other app characteristics.

### Strengths and Limitations

While previous studies have examined the type of alcohol-related apps that are available, this is the first to our knowledge to have examined the BCTs present in alcohol apps with an alcohol reduction focus. Documenting their content allows researchers to refine their future evaluations in terms of active ingredients and may help users to be better informed. It may also allow for future regulation of apps to be facilitated [23,24].

This study has several limitations. First, the presence of BCTs was coded but not their “dose” [56], that is, their intensity and whether or how often it was repeated, nor the quality with which it was delivered [57], which is likely to influence the degree with which it was engaged with by users. Engagement with a BCT is important if behavior change is to occur, but many digital interventions experience high levels of attrition [58]; more understanding of the ways in which users engage with mHealth apps is required. Second, the mHealth market is constantly evolving. New apps are added on a regular basis, and both Google and Apple frequently change their algorithms for returning search results. Therefore, these findings should be seen as representing a snapshot in time. Finally, the BCTs were identified by a taxonomy developed for face-to-face rather than digital interventions [30]. While an acceptable interrater reliability was established, the list may not be exhaustive and a similar method designed specifically for digital interventions is needed.

### Conclusions

While a minority of alcohol-related apps promoted health, the majority implicitly or explicitly promoted the use of alcohol. Alcohol-related apps that focused on alcohol reduction usually contained few BCTs or few BCTs frequently found in other interventions, and their popularity or user ratings were only weakly related to their BCT content. None of the apps mentioned theory, and the few apps that mentioned evidence usually referred to evidence about guidelines. The popularity of these apps suggests that users may value content that makes explicit reference to evidence.

## Multimedia Appendix 1

Taxonomy of 42 BCTs used to treat excessive alcohol use. Tailored for apps.

## Abbreviations

BAC: blood alcohol content
BCT: behavior change technique
PABAK: prevalance and bias adjusted kappa
