# Supplementary material for: Behavior Change Techniques in Popular Alcohol Reduction Apps: Content Analysis
Source: J Med Internet Res. 2015 May 14;17(5):e118. doi: 10.2196/jmir.4060 (PMC4468601; doi:10.2196/jmir.4060)
Supplement: Supplementary file 1 [file jmir_v17i5e118_app1.pdf]

Taxonomy of 42 BCTs used to treat excessive alcohol use identified in 9 clinical guidance/self help documents and 3 service manuals

Tailored for apps.

1. **Provide information on consequences of excessive alcohol use and drinking cessation**
Give, or make more salient, information about the harm caused by excessive drinking and the benefits of reducing excessive alcohol consumption.

2. **Boost motivation and self efficacy**
Give encouragement and bolster confidence in the ability to reduce excessive alcohol use.

3. **Provide feedback on performance**
Give information on progress towards reducing excessive alcohol use. For example, graphs or other information that indicate how consumption has changed over time.

4. **Provide rewards contingent on successfully reducing excessive alcohol use/abstaining**
Give praise or other rewards if the user has cut down on their drinking.

5. **Provide normative information about others' behaviour and experiences**
Give information about how the user's experience compares with other people's experiences.

6. **Prompt commitment from the user there and then**
Encourage the user to affirm or reaffirm a strong commitment to start, continue or restart the attempt to reduce excessive alcohol use. Requires interaction from the user – i.e. by entering information into a field, clicking a button etc. Simple presentation of information is insufficient.

7. **Provide rewards contingent on effort or progress**
Give praise or other rewards for the effort the user is making.

8. **Identify reasons for wanting and not wanting to reduce excessive alcohol use**
Help the user to arrive at a clear understanding of his or her feelings about reducing excessive alcohol use, why it is important to reduce and any conflicting motivations. Requires interaction from the user – i.e. by entering

information into a field, selecting items from a list etc. Simple presentation of information is insufficient.

9. Explain the importance of abrupt cessation

Explain why it is better to stop abruptly rather than cut down gradually if at all possible.

10. Facilitate barrier identification and problem solving

Help the user to identify general barriers (e.g. susceptibility to stress) that might make it harder to reduce excessive consumption/abstain and develop ways of addressing these.

11. Facilitate relapse prevention and coping

Help the user understand how lapses occur and how they lead to relapse and to develop general strategies for preventing lapses or avoiding lapses turning into relapse.

12. Facilitate action planning/know how to help identify relapse triggers

Help the user identify specific triggers that generate urge/want/need to drink and develop and reinforce plans for avoiding these or coping with the motivation to drink when it occurs. Code 1 if only information provided, 2 if interaction sought from the user – i.e. by choosing triggers from a list or specifying one(s) of their own, and then developing a plan for dealing with these triggers.

13. Facilitate goal setting

Help the user to set goals that support the aim of reducing their drinking.

14. Prompt review of goals

Review how far the user has achieved the main goal of reducing excessive consumption/abstinence and any other goals that are supportive of it (e.g. putting in place plans to avoid triggers). For example, text or graphical feedback that indicates how levels of current consumption relate to levels of previous consumption.

15. Facilitate self-recording

Allow the user to record details of current and past consumption and when urges to drink are strong and less strong.

16. Change routine

Advise on ways of changing daily or weekly routines to minimise exposure to drinking cues.

17. Advise on environmental restructuring

Advise on ways of changing the physical environment to minimise exposure to drinking cues (e.g. removing bottles from the house).

18. Set graded tasks
Set small achievable goals where appropriate (e.g. take one day at time).

19. Advise on conserving mental resources
Advise on ways of minimising stress and other demands on mental resources (activities that require mental effort).

20. Advise on avoidance of social cues for drinking
Give specific advice on how to avoid being exposed to social cues for drinking (e.g. explaining to friends that you have stopped).

21. Advise on/facilitate use of social support
Advise on or facilitate development of social support from friends, relatives, colleagues or 'buddies'.

22. Give options for additional and later support
Provide links to additional and later support where these are available (e.g. websites, self help groups, telephone helpline).

23. Tailor interactions appropriately
Use relevant information from the user to tailor the behavioural support provided.

24. Emphasise choice
Emphasise user choice within the bounds of evidence based practice.

25. Assess current and past drinking behaviour
Assess amount drunk, age when started, pattern of drinking behaviour. Includes consumption measures such as AUDIT, FAST or others.

26. Assess current readiness and ability to reduce excessive alcohol use
Assess current level of motivation to reduce excessive alcohol use and confidence in success. Requires interaction from the user, for example by answering questions about their level of motivation/confidence in success.

27. Assess past history of attempts to reduce excessive alcohol use
Assess number and duration of past attempts and experiences related to these, including factors that led to drinking.

28. Assess withdrawal symptoms
Assess the presence and severity of alcohol withdrawal signs and symptoms.

29. Build general rapport
Establish a positive, friendly but professional relationship with the user and foster a sense that the user's experiences are understood.

30. Elicit and answer questions
Prompt questions from the user and answer clearly and accurately. Requires input from the user and responses from an expert.

31. Explain expectations regarding treatment programme
Explain to the user the treatment programme, what it involves, the active ingredients and what it requires of the drinker.

32. Offer/direct towards appropriate written materials
Distinguish what are and are not, appropriate written materials and offer/direct/link users to these in ways that promotes their effective use. Differs from BCT22 in that it requires editorial guidance from the app as to what is and is not appropriate. If links are present without guidance, code BCT22.

33. Provide information on withdrawal symptoms
Describe to drinkers what are, and are not, alcohol withdrawal symptoms, how common they are, how long they typically last, what causes them and what can be done to alleviate them.

34. Use reflective listening
Adopt a style of interaction that involves listening carefully to the user and where appropriate reflecting back to the user key elements of what s/he is saying. Requires the app to include a communication element that connects them with an expert.

35. **Elicit user views**
Prompt the user to give views on drinking, reducing excessive alcohol use/abstaining and any aspect of the behavioural support programme. Requires interaction from the user – i.e. by entering information into a field about their views on drinking or the support programme, selecting items from a list etc.

36. **Summarise information/confirm user decisions**
Provide a summary of information exchanged and establish a clear confirmation of decisions made and commitments entered into.

37. Provide reassurance

Give general reassurance to the user that his/her experiences are normal and time limited, and provide positive expectations of success based on experience with other drinkers in the same situation.

38. Model/ demonstrate the behaviour

Involves showing the person how to correctly perform a behaviour e.g., through physical or visual demonstrations of behavioural performance, in person or remotely.

39. Prompt use of imagery

Teach the person to imagine successfully performing the behaviour or to imagine finding it easy to perform the behaviour, including component or easy versions of the behaviour.

40. Motivational Interviewing

This is a specific set of techniques involving prompting the user to provide self motivating statements and evaluations of own behaviour to minimise resistance to change (includes motivational counselling). Normally this technique will be mentioned by name. Only rate this technique if explicitly referred to by name, not if one identifies specific elements of it.

41. General communication skills training

This includes any technique directed at general communication skills but not directed towards a particular behaviour change. Often this may include role play and group work focusing on listening skills or assertive skills..

42. Behaviour substitution

Substituting the undesired behaviour or its associated activities with a behaviour that does not promote excessive alcohol use e.g. substituting going to the pub with going to the cinema.
